# Molecular and morphological evidence for recognition of two species within *Harpagonella* (Amsinckiinae, Boraginaceae)

**DOI:** 10.3897/phytokeys.70.9053

**Published:** 2016-09-20

**Authors:** C. Matt Guilliams, Timothy Jang, Bruce G. Baldwin

**Affiliations:** 1 Jepson Herbarium and Department of Integrative Biology, University of California Berkeley, 1001 Valley Life Sciences Building #2465, Berkeley CA 94720-2465, United States of America; 2 Santa Barbara Botanic Garden 1212 Mission Canyon Road, Santa Barbara, CA 93105-2126, United States of America

**Keywords:** Amsinckiinae, Boraginaceae, Harpagonella, Pectocarya

## Abstract

Recent taxonomic treatments of the genus *Harpagonella* have included only one lower taxon, *Harpagonella palmeri* A. Gray. However, a larger-fruited variety of *Harpagonella palmeri* from Arizona and Sonora was described by I.M. Johnston in 1924. He continued to recognize this taxon – Harpagonella palmeri var. arizonica – in his treatment of the genus in Kearney and Peebles’s Arizona Flora in 1960. Here, we provide two lines of molecular evidence and quantitative morphological evidence from calyx characters showing that plants of *Harpagonella* from Arizona, Sonora, and central Baja California, corresponding to Johnston’s var. arizonica, are distinct from *Harpagonella palmeri* of southern California and Baja California. We make the new combination *Harpagonella arizonica* (I.M. Johnston) Guilliams & B.G. Baldwin, **comb. nov.** for the plants from Arizona, Sonora, and central Baja California.

## Introduction

**Figure 1.**
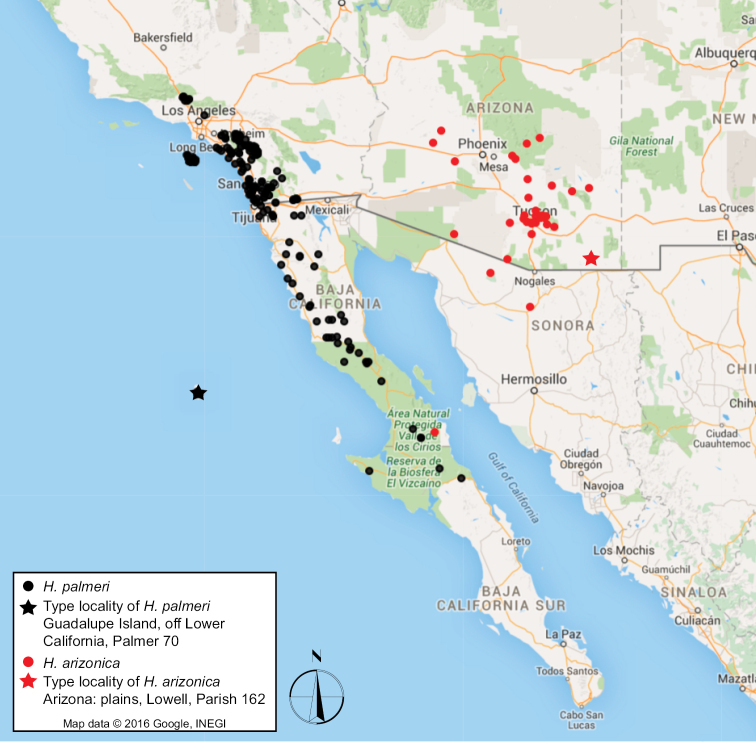
Map of western North America showing *Harpagonella* collections in major herbaria based on available specimen data from GBIF and Bajaflora. Type collection localities are indicated with black star for *Harpagonella palmeri* and a red star for *Harpagonella arizonica*.

*Harpagonella* A. Gray is a genus of Boraginaceae, subtribe Amsinckiinae (see Chacón et al. 2016 and Luebert et al. 2016) that occurs disjunctly in western North America, with populations in southern California, USA, and adjacent Baja California, México and other populations in southern Arizona, USA, and adjacent northwestern Sonora, México (Figure 1). The only species recognized in the genus, *Harpagonella palmeri* A. Gray, was described in 1876 from an 1875 collection by Edward Palmer on Guadalupe Island, Baja California. In 1924, Ivan M. Johnston recognized two varieties in *Harpagonella palmeri*, var. arizonica and var. palmeri. The former taxon, then known from Arizona and adjacent Sonora, was said to differ from var. palmeri, of California and Baja California, in having longer “cornute processes on the fruiting calyx” and larger nutlets (Johnston 1924). Furthermore, the plants of California and Baja California are often found on clayey soils, while those of Arizona and Sonora often occur in sandy or gravelly soils. In his treatment of the Boraginaceae for the Arizona Flora (Kearney and Peebles 1960), Johnston retained the taxon as a variety, but most other treatments of the genus recognize *Harpagonella palmeri* without varieties (e.g., Munz 1973, Veno 1979, Kelley and Messick 2014).

**Figure 2.**
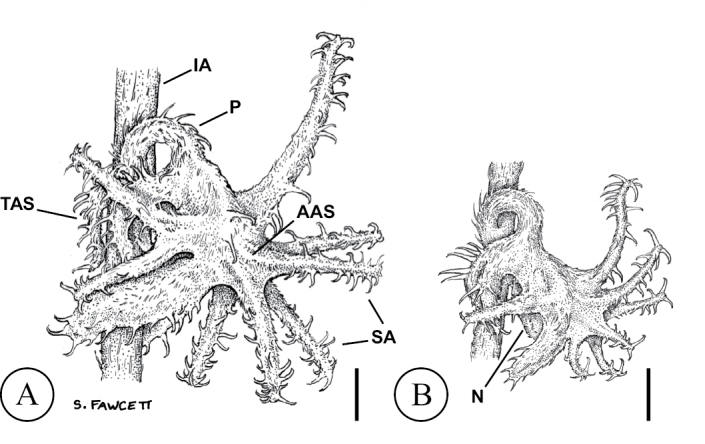
Fruits of *Harpagonella* in lateral view, from A) southern Arizona (*Tedford 1043*, ARIZ403065) and B) southern California (*Bramlet 2301*, ARIZ345225). Although morphologically similar, note overall difference in size. Scale bars are each approximately 1 mm. Labels: (AAS) sepals away from inflorescence axis in flower; (IA) inflorescence axis; (N) nutlet; (P) pedicel; (SA) sepal appendages; (TAS) sepals toward inflorescence axis in flower.

*Harpagonella* has been regarded as the most morphologically distinctive member of the Amsinckiinae, largely because of ornamentation of the calyx in fruit that is unique to the genus (Johnston 1924, Veno 1979). The genus was placed in its own tribe, Harpagonelleae, for this reason (Gürke 1897). In *Harpagonella*, the calyx is pentamerous, with the two sepals away from the inflorescence axis connate for >80% of their length and the three other sepals free while in flower. The two fused sepals are strongly accrescent, becoming conduplicate, indurate, and often more or less enveloping one nutlet or sometimes both nutlets at fruit maturity (Figure 2). As the fruit matures, five to ten subterete appendages with distal retrorse barbs develop on the pair of fused sepals, giving the fruit the appearance and function of a grappling hook, which is the common name for the genus. The pedicel is also accrescent. It recurves or rarely coils as the fruit matures, placing the lobes of the fused sepals against the inflorescence axis. As Gray (1876) noted, these modifications effectively result in the transfer of dispersal function from the nutlet, as is typical in many Amsinckiinae, to the calyx. The gynoecium in *Harpagonella* is also distinctive. It has been reduced from the typical condition in the Amsinckiinae of four ovules and a fruit of four nutlets to two developing ovules and two nutlets, with the other two ovules early abortive. Unlike the nutlets of many close relatives, e.g. *Pectocarya*, the two nutlets of *Harpagonella* are largely without ornamentation, bearing only short hairs.

We included *Harpagonella* in broad phylogenetic and taxonomic studies of some members of the Boraginaceae subtribe Amsinckiinae (Guilliams 2015). During the phylogenetic study, we included several samples of *Harpagonella palmeri* from throughout its range with the goal of evaluating phylogenetic structure of the included samples, with attention to historical taxonomy. We also examined herbarium sheets representing both previously recognized varieties of *Harpagonella palmeri*, taking measurements of the calyx appendages and overall size of the fruit. Although a full phylogenetic study will be published later, we present the results of this study here in reduced form so that the resulting new combination can be available for use in the treatment of *Harpagonella* for the Flora of North America, North of México.

## Methods

### Phylogenetic analyses

DNA was extracted from 12 samples of *Harpagonella* and 2 samples of *Pectocarya* using a modified CTAB protocol (Doyle and Doyle 1987). Samples included in this analysis are given in Table 1 and were selected on the basis of geographic distribution of the two putative taxa and recency of collection. Six of these samples were from Arizona and were morphologically consistent with Harpagonella palmeri var. arizonica sensu Johnston (1924). The other six samples were from California and adjacent Baja California and were morphologically consistent with Harpagonella palmeri var. palmeri. One sample each of Pectocarya linearis DC. var. ferocula I.M. Johnst. and *Pectocarya recurvata* I.M. Johnst. were included as outgroup taxa.

**Table 1.** Specimens of *Harpagonella* and outgroups used in phylogenetic analysis, including collector and collection numbers, herbarium accession numbers, and GenBank accession numbers by DNA region.

Taxon	Collector and Collection Number	Herbarium Accession Number	GenBank Accession Numbers					
			ITS	ETS	rpL16	rps16	trnK-rps16	trnL-trnF
Harpagonella palmeri var. arizonica	J.E. Bowers 2395	ARIZ241135	KX151054	–	KX151070	KX151084	KX151098	KX151108
	T.R. Van Devender 88-54	ARIZ278363	KX151052	KX151044	KX151068	KX151082	KX151096	KX151106
	S.P. McLaughlin & J.E. Bowers 4476	ARIZ307288	KX151053	–	KX151069	KX151083	KX151097	KX151107
	A.L. Reina G. & T.R. Van Devender 2003-194	ARIZ364715	KX151056	–	KX151072	KX151086	KX151100	KX151110
	T.R. Van Devender & A.L. Reina G. 2005-842	ARIZ377143	KX151055	–	KX151071	KX151085	KX151099	KX151109
	J. Tedford 599	ARIZ388168	KX151051	KX151043	KX151067	KX151081	KX151095	KX151105
Harpagonella palmeri var. palmeri	C.M. Guilliams 1414	n/a	KX151057	KX151045	KX151073	KX151087	KX151101	KX151113
	C.M. Guilliams 1421	n/a	KX151058	KX151046	KX151076	KX151088	KX151102	KX151114
	J.P. Rebman 8348	UC1790083	KX151059	KX151047	KX151075	KX151089	KX151103	KX151111
	S. Boyd & T.S. Ross 7906	UC1871078	KX151062	–	KX151078	KX151092	–	KX151116
	S. Boyd & T.S. Ross 8212	UC1871288	KX151061	–	KX151077	KX151091	–	KX151115
	J.P. Rebman 8031	UC1790065	KX151060	KX151048	KX151074	KX151090	KX151104	KX151112
*Pectocarya penicillata*	R.B. Kelley 1968	n/a	KX151063	KX151049	KX151065	KX151079	KX151093	KX151117
*Pectocarya platycarpa*	R.B. Kelley 1983	n/a	KX151064	KX151050	KX151066	KX151080	KX151094	KX151118

Polymerase chain reaction (PCR) was used to amplify the internal transcribed spacer (ITS) and the external transcribed spacer (ETS) of nuclear ribosomal DNA, and the *rpl16*, *rps16*, *trnK-rps16*, and *trnL-trnF* regions of the chloroplast genome. All PCR reactions except for those targeting the ETS region were performed using previously published primers and reaction conditions (see Baldwin et al. 1995, Shaw et al. 2005, Shaw et al. 2007). The 5’ ETS primer was designed following the protocol of Baldwin and Markos (1998). PCR products were cleaned using USB ExoSAP-IT (Affymetrix, Santa Clara, CA, USA) using the standard protocol. Bidirectional sequencing was performed on an Applied Biosystems 3730xl DNA Analyzer at the Barker DNA Sequencing core facility at UC Berkeley. Contigs were assembled and edited in Geneious R6 (Drummond et al. 2013). Sequences were initially aligned under the default parameters using the Geneious alignment tool in Geneious, then further refined by hand.

For each DNA region, models of sequence evolution were estimated using jModelTest (Posada 2008). Bayesian phylogenetic analyses were performed and summarized using the BEAST suite of programs. Four separate analyses of 10 million generations were performed in BEAST v.1.7.4 (Drummond and Rambaut 2007), with the first 25% of trees discarded as burn-in. Convergence was assessed using Tracer v.1.7.4 (Rambaut and Drummond 2007). Post burn-in runs were combined using Log Combiner v.1.7.4. The maximum clade credibility tree (MCCT) was found and clade credibility values calculated using Tree Annotator v.1.7.4.

Separate maximum likelihood analyses for nrDNA and cpDNA were performed using RAxML v1 plug-in in Geneious v8.1.8 (Drummond AJ et al. 2015). Maximum likelihood bootstrap values resulting from these analyses were added to the MCCT.

### Morphological analyses

Morphological data were taken from a total of 32 physical specimens of Harpagonella palmeri var. arizonica and 27 physical specimens of Harpagonella palmeri var. palmeri. Physical specimens measured were those available from the ARIZ, JEPS, and UC herbaria with mature fruits. We also measured high quality digital scans of type material of both taxa. For each specimen, we measured and averaged values from up to five fruits for maximum fruit length along an axis oriented from the pedicel base to the most distant point (including subterete appendages; mm), maximum fruit width along an axis perpendicular to maximum fruit length (including subterete appendages; mm), and maximum length of subterete appendages (mm). Measurements of physical specimens were taken with a digital caliper to the nearest hundredth of a millimeter. Measurement of digital specimens were made in ImageJ (Abramoff MD et al. 2004). Nutlet length has been reported as different between the two varieties, but measuring this feature would have required occasional destructive sampling and was therefore avoided.

Morphological data were explored using boxplots and basic descriptive statistics. Student’s t-tests were performed to evaluate the statistical significance of the differences between the varieties for the features measured. All statistical analyses were performed in R (R Development Core Team 2008).

## Results

### Phylogenetic patterns in *Harpagonella*

The nuclear dataset comprising ITS and ETS was 1,082 total bases in length. For these loci, jModelTest determined a best-fit model of sequence evolution of GTR+I. In the matrix, 79 positions were variable and phylogenetically informative, 29 were variable and not phylogenetically informative, and 974 were invariant.

**Figures 3.**
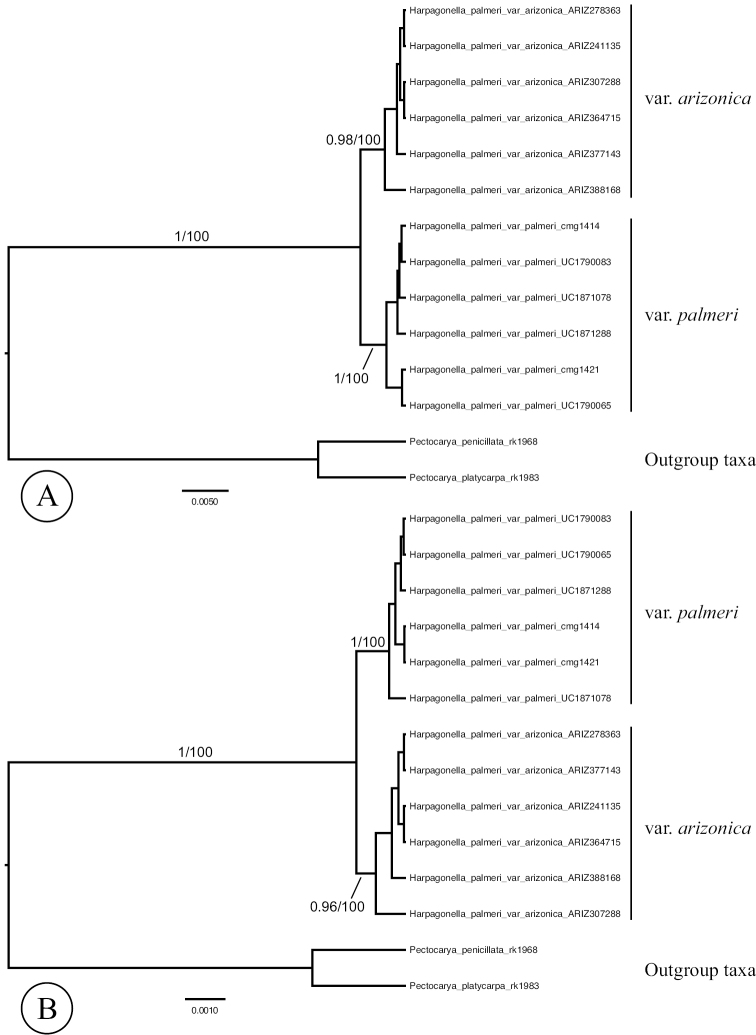
Maximum clade credibility trees from phylogenetic analysis of the: **A** combined, partitioned nuclear DNA regions, and **B** combined, partitioned chloroplast DNA regions. Values on branches are Bayesian posterior probabilities followed by maximum likelihood bootstrap values.

The MCCT resulting from the analysis of the concatenated nuclear DNA matrix is given in Figure 3A. Samples of each variety of *Harpagonella* are reciprocally monophyletic and clades by taxon are strongly supported. The clade of samples of var. arizonica was supported with a posterior probability of 0.98 and a maximum likelihood bootstrap value of 100. The clade of samples of var. palmeri was supported with a posterior probability of 1 and a maximum likelihood bootstrap value of 100. Support for phylogenetic relationships within each clade was poor.

The chloroplast dataset comprising *rpl16*, *rps16*, *trnK-rps16*, and *trnL-trnF* was 3,442 total bases in length. For these loci, jModelTest determined a best-fit model of sequence evolution of GTR+I. Of these, 51 positions were variable and phylogenetically informative, 30 were variable and not phylogenetically informative, and 3,361 were invariant.

The MCCT resulting from the analysis of the concatenated chloroplast DNA matrix is given in Figure 3B. Samples of each variety of *Harpagonella* are reciprocally monophyletic and clades by taxon are strongly supported. The clade of samples of var. arizonica was supported with a posterior probability of 0.96, and a maximum likelihood bootstrap value of 100. The clade of samples of var. palmeri was supported with a posterior probability of 1 and a maximum likelihood bootstrap value of 100. Support for phylogenetic relationships within each clade was poor.

The split between *Harpagonella* and outgroup sequences as well as the branches subtending varieties of *Harpagonella palmeri* were all supported by a number of shared nucleotide substitutions as well as insertion/deletions (indels). The *Harpagonella*-outgroup split was supported by 68 substitutions in the nuclear dataset, and 46 substitutions and 31 indels in the chloroplast dataset. The branch subtending the clade of var. arizonica samples was supported by 4 nucleotide substitutions in the nuclear dataset, and 1 substitution and 5 separate indels in the chloroplast dataset. The branch subtending the clade of var. palmeri samples was supported by 3 nucleotide substitutions in the nuclear dataset and 3 substitutions in the chloroplast dataset.

### Morphological patterns in *Harpagonella*

**Figure 4.**
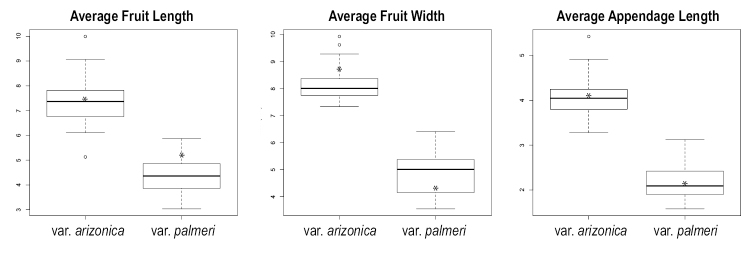
Box and whisker plots by taxon of **A** average maximum fruit length (mm), **B** average maximum fruit width (mm), **C** average maximum subterete appendage length (mm). Asterisks denote the measured values of type specimens. Note significant differentiation in all features measured.

Harpagonella palmeri var. arizonica and Harpagonella palmeri var. palmeri differ in all three features measured and the differences are highly significant statistically (p << 0.001). Box and whisker plots of the measured morphological features are presented in Figure 4. Values for measurements of type specimens are denoted by an asterisk. Average maximum fruit length ranged from 5.13 to 9.99 mm (average = 7.38 mm; type = 7.58 mm) in Harpagonella palmeri var. arizonica and from 3.04 to 5.87 mm (average = 4.38 mm; type = 5.38 mm) in Harpagonella palmeri var. palmeri (t = 14.027, df = 55.488, p < 2.2 × 10^-16^). Average maximum fruit width ranged from 7.33 to 9.33 mm (average = 8.17 mm; type = 8.88 mm) in Harpagonella palmeri var. arizonica and from 3.55 to 6.41 mm (average = 4.84 mm; type = 4.33 mm) in Harpagonella palmeri var. palmeri (t = 17.912, df = 49.56, p < 2.2 × 10^-16^). Average maximum subterete appendage length ranged from 3.28 to 5.42 mm (average = 4.08 mm; type = 4.12 mm) in Harpagonella palmeri var. arizonica and from 1.58 to 3.12 mm (average = 2.19 mm; type = 2.10 mm) in Harpagonella palmeri var. palmeri (t = 16.767, df = 55.976, p < 2.2 × 10^-16^).

## Discussion

The separate phylogenetic analyses of nrDNA and cpDNA presented here each recover two clades within *Harpagonella palmeri* corresponding to the two named varieties. Statistical support for these groupings was very high, with posterior probabilities above 0.96 and maximum likelihood bootstrap values of 100 in all cases. The *Harpagonella*-outgroup split as well as clades of samples by variety were each supported by numerous nucleotide substitutions and indels. We take this as strong evidence for two evolutionary lineages in the genus.

Morphologically, these two lineages differ in all measured aspects of fruit size. Plants primarily from Arizona and Sonora are significantly larger in maximum fruit length, maximum fruit width, and appendage length. Box and whisker plots for these features show that the ranges of measurements of these characters between the two lineages are mostly non-overlapping. Although unmeasured here, nutlet size in *Harpagonella* was suggested by Johnston (1924) to be larger in plants from Arizona and Sonora than in plants from California and Baja California. These differences are quantitative, not qualitative, and absent a formal statistical analysis of morphology, Veno (1979) advocated for recognizing no infraspecific taxa in *Harpagonella palmeri*, stating that “this feature is variable and somewhat clinal, and does not provide a significant or reliable basis for taxonomic delimitation.” The data presented here suggest instead that these quantitative characters appear to be sufficient for reliable delimitation of two taxa corresponding to the evolutionary lineages recovered in the phylogenetic analysis.

Herbarium study of 366 specimens representing 291 gatherings of *Harpagonella* has permitted the evaluation of the geographic range of these morphologically distinct evolutionary lineages, which is especially critical for specimens collected on the Baja California Peninsula, where both named varieties have been reported. Specimens of plants with larger fruits corresponding to Johnston’s Harpagonella palmeri var. arizonica are almost entirely from Arizona and Sonora, with two collections attributable to this taxon made from desert regions of Baja California at mid-peninsula (*Moran 12682*, 28.29007, -113.12146; *Moran 12845*, 28.28333, -113.65). We have observed and confirmed the taxonomic identity of a specimen of the former (DS598325) but not the latter. Specimens of plants with smaller fruits corresponding to Johnston’s concept for Harpagonella palmeri var. palmeri are known primarily from southwestern California and the adjacent western coastal areas of the Baja California Peninsula, with collections ranging as far to the south as the Vizcaino Peninsula on the Pacific Coast in Baja California Sur.

The biogeographic pattern displayed by *Harpagonella* – a disjunction between the California Floristic Province sensu Howell (1957) and central, southern Arizona and adjacent Sonora – is somewhat common yet underexplored. Raven and Axelrod (1978) describe this pattern briefly in their important paper on the origin of the California flora, and provide a table of 35 genera, species, or species pairs that have this pattern. To their list of taxa, we add *Harpagonella* based on evidence presented here.

## Taxonomic treatment

Based on complete and well-supported reciprocal monophyly in two unlinked genomic partitions, statistically significant morphological differences, and essentially non-overlapping geographic ranges, the two lineages of *Harpagonella* resolved here merit recognition at the species level under the criteria of phylogenetic species concepts (see Mishler and Theriot 2000) as well as longstanding taxonomic practice. To recognize a taxon at species rank for the large-fruited plants found primarily in the deserts of Arizona and Sonora, the following new combination is needed.

### Harpagonella arizonica

Taxon classificationPlantaeBoraginalesBoraginaceae

(I.M. Johnston) Guilliams & B.G. Baldwin comb. nov.

urn:lsid:ipni.org:names:77157712-1

#### BASIONYM.

Harpagonella palmeri A. Gray var. arizonica I.M. Johnston. Contr. Gray Herb. 73: 75. 1924. TYPE: U.S.A. Arizona: “plains, Lowell,” *W.F. Parish 162*, May 3, 1884, (holotype: GH! digital image).

#### SPECIMENS EXAMINED.

Specimens listed alphanumerically by collector within a region. (*=specimen measured; è =specimen also used in molecular study; **bold**=type specimen) ***Harpagonella arizonica*: MÉXICO. Baja California.** *Moran 12682* (DS). **Sonora.** *Keck 3963* (DS, POM), *Reina & Van Devender 2003-194*è (ARIZ, ASU), *Van Devender 2005-842*è (ARIZ). **UNITED STATES. Arizona.** *Abrams 12944* (DS), *Baker 8203* (ASU), *Baker 15963* (ASU), *Barr 67-78* (ASU), Barr 67-82* (ARIZ, ASU), *Benson 9302* (POM), Bingham 527* (ARIZ), *Bingham 1402* (ASU), Bowers 2250* (ARIZ), Bowers 2280* (ARIZ), Bowers 2395*è (ARIZ), Bowers 2461* (ARIZ), *Boyle 8026* (ARIZ), *Brandegee, T.S. s.n. 19 April 1889* (UC), *Butterwick 4349* (ASU), *Butterwick 4550* (ASU), *Butterwick & Hillyard 5793* (ARIZ, ASU), *Butterwick 7419* (ASU), *Carter s.n. 17 March 1936* (ARIZ), *Cave 16* (ARIZ), *Damrel 1618-B8* (ASU), *Daniel 2581* (ASU), *Daniel & Butterwick 3853* (CAS), *Daniel 3907* (ASU), *Doan 441* (ASU), *Ducote 683* (ASU), *Eastwood 8130* (CAS), *Farruggia 1832* (ASU), *Felger 05-218* (ASU), *Fosberg 10605* (CAS, RSA), *Fosberg 10664* (CAS, POM), *Freeman* (ASU), *Gillespie 5429* (DS), Griffiths s.n. date unknown* (ARIZ), *Halse 1701* (CAS), *Halverson 379* (ASU), *Harrison & Fulton 6608* (POM), *Harrison & Kearney 6654* (POM), *Higgins 6480* (ASU), *Hitchcock 25598* (DS, RSA), *Imdorf & Rice 427* (ASU, ARIZ), *Imdorf 587* (ASU), Kearney 6654* (ARIZ), *Keck 2998* (DS), *Keil 1051* (ASU), *Keil 1484* (ASU), *Keil 2864* (ASU), *Keil 4082* (ASU), *Keil 4168* (ASU), *Keil K-11216* (ASU), *Landrum 6656* (ASU), *Landrum 11176* (ASU), *Lane 1035* (ASU), *Lane 1067* (ASU), *Lehto 181* (ASU), *Lehto 307* (ASU), *Lehto 1648* (ASU), *Lehto 1652* (ASU), *Lehto 4594* (ASU), *Lehto 7766* (ASU), *Lehto 10374* (ASU), *Lehto 10389* (ASU), *Lehto 10408* (ASU), *Lehto 10687* (ASU), *Lehto 11733* (ASU), *Lehto 17494* (ASU), *Lehto 17504* (ASU), *Lehto 17541* (ASU), *Lehto 12874-b* (ASU), *Lehto L-19732* (ASU), *Lehto L-19740* (ASU), *Makings 2018* (ASU), *Makings, L. Fertig, & W. Fertig 4346* (ASU, RSA), *Manton 236* (ASU), Mason 1663* (ARIZ, CAS), *Mauz, Rosen, & Rautenkranz 2005-19* (ARIZ), *McGill LAM1280* (ASU, RSA), McLaughlin 4476*è (ARIZ), *Orcutt 173* (CAS), *Parfitt 2498* (ASU), ***Parish 162* (GH; holotype)**, *Parish s.n. 1909* (DS), *Pase 1599* (ASU), Peebles 1426* (ARIZ), Peebles 3693* (ARIZ), *Pierce 296* (ASU), *Pinkava 4672* (ASU), *Pinkava 10122* (ASU), *Pinkava 10261* (ASU), *Pinkava 10893* (ASU), *Pinkava 11655* (ASU), *Price 829* (ASU), *Rand 15* (ASU), *Rand 152* (ASU), *Reeves 6447-a* (ASU), *Reina & Van Devender 97-269* (ARIZ), *Rice 328* (ASU), *Rice 1121* (ASU), *Rice 1586-a* (ASU), *Rice 1598* (ASU), *Jones, S. 1433* (ASU), *Schramm, Bond, & Bond 9* (ASU, RSA), *Shreve 7497* (ARIZ), Shreve 10113* (ARIZ, DS), *Smith 1577* (ASU), *Swingle s.n. 1914* (ARIZ), Tedford 582* (ARIZ), Tedford 599*è (ARIZ), *Tedford 614* (ARIZ), Tedford & Rose 1034* (ARIZ), Thornber 2562* (ASU, ARIZ, CAS, RSA), Thornber 2581* (ARIZ, CAS, RSA), *Thornber 4683* (ARIZ), Thornber 5488* (ARIZ), Thornber s.n. 1905* (ARIZ), Thornber s.n. 1913* (ARIZ), Toumey 5014* (ARIZ), Turner 78-41* (ARIZ), VanDevender 88-54*è (ARIZ), Van Devender 2003-23* (ASU, ARIZ), *W. Fertig, Makings, & Alcock 29265* (ASU), Warren 68-25* (ARIZ), Warren 68-51* (ARIZ), Wiggins 8420* (ARIZ), *Wiggins 8690* (DS), *Wood* (ASU). ***Harpagonella palmeri*: MÉXICO. Baja California.** *Bacigalupi 3067* (DS, RSA, UC), Boyd 5319* (RSA, UC), *Boyd & Ross 5464* (RSA), *Boyd & Ross 5761* (RSA), *Boyd, Gross, O’Brien, & Hamilton 10352* (RSA), *Breedlove 62271* (CAS, RSA), *Carter, Chisaki, & Moran 1056* (UC), Dressler 668* (ARIZ), *Epling & Stewart s.n. 9 April 1936* (DS), *Haines & Stewart s.n. 7 February 1935* (DS), *Howell 8306* (CAS), *Jones, M.E. s.n. 11 April 1882* (POM), *Moran 6562* (POM), *Moran 6677* (DS), *Moran 6750* (DS, RSA), *Moran 12770* (UC), *Moran 19378* (CAS), *Moran 19992* (POM), *Porter 10551* (RSA), *Rebman & Delgadillo 1638* (ASU), *Rebman & Roberts 4856* (ASU), *Sanders, Rodriguez, West, et al. 5466* (ASU), *Thomas 15730* (DS), *Thorne, Liston, Mistretta 62122* (RSA), *Van Devender 91-348* (ARIZ), *Van Devender, T.R. & R.K. Van Devender 91-239* (ARIZ), *Wiggins & Ernst 12* (UC), *Wiggins & Thomas 67* (CAS), *Wiggins & Ernst 120* (DS), *Wiggins 4265* (DS, POM), *Wiggins 4415* (POM), *Wiggins 4463* (DS, POM), *Wiggins 7600* (DS, UC). **UNITED STATES. California.** Atwood 17833* (UC), Bacigalupi 8261* (JEPS), *Banks & Boyd 57* (RSA), *Banks & Boyd 316* (RSA), *Banks & Boyd 398* (RSA), *Banks 1652* (RSA), *Banks 1680* (RSA), *Bell, Clark, Goss, Green, & Rusiniak 3546* (RSA), *Boyd 1384* (ARIZ, CAS, RSA), *Boyd 1396* (CAS, RSA), *Boyd 1399* (CAS, RSA), Boyd 1589* (ARIZ, CAS, RSA), Boyd 1644* (ARIZ, CAS, RSA), Boyd 1767* (ARIZ, CAS, RSA), Boyd 1790* (ARIZ, CAS, RSA), Boyd 1816* (CAS, RSA, UC), Boyd 3045* (UC), *Boyd, Ross, & Arnseth 3029* (RSA), *Boyd, Ross, & Arnseth 3036* (RSA), *Boyd, Ross, & Arnseth 3045* (RSA), *Boyd, Ross, & Arnseth 3116* (RSA), *Boyd, Ross, & Arnseth 3133* (RSA), *Boyd, Ross, & Arnseth 3196* (RSA), Boyd, Ross, & Arnseth 3206* (RSA, UC), *Boyd, Ross, & Arnseth 3920* (RSA), *Boyd, Ross, Arnseth, & Bonilla 4008* (RSA), *Boyd, Ross, Arnseth, & Bonilla 4060* (CAS, RSA), *Boyd, Ross, Arnseth, & Bonilla 4110* (RSA), *Boyd, Arnseth, Rasmussen, & Cota 4605* (RSA), *Boyd 6165* (RSA), *Boyd & Mistretta 6311* (RSA), *Boyd 6901* (RSA), *Boyd 6962* (RSA), *Boyd & Ross 7302* (RSA), *Boyd & Ross 7906*è (RSA, UC), *Boyd & Ross 8212*è (RSA, SBBG, UC), *Boyd & Ross 8220* (RSA), *Boyd & Ross 8244* (RSA), Boyd & Ross 8249* (ARIZ, RSA), *Boyd & Banks 8279* (RSA), *Boyd 10414* (RSA, UC), *Boyd s.n. 28 March 1982* (RSA), *Boyd s.n. 27 April 1982* (RSA), Bramlet 2301* (ARIZ), *Bramlet 2370* (CAS), *Bramlet 2394* (RSA), *Bramlet 2399* (RSA), *Bramlet & Coleman 2418* (RSA), *Bramlet 2982* (RSA), *Bramlet 2988* (RSA), *Bramlet 3352B* (RSA), Brandegee T.S. 824* (CAS, POM, UC), *Brandegee s.n. 12 April 1894* (DS), *Brandegee s.n. 15 April 1894* * (RSA, UC), Brandegee T.S. s.n. 8 April 1895* (UC), Gander 1128* (DS, POM, UC), Gander 3112* (JEPS), Gander 5072* (JEPS, RSA, UC), *Grant 5218* (DS), *Grant & Wheeler 540* (UC), *Gross, Fraga, Virgen, Thibault 1781* (RSA), *Gross, Fraga, Virgen, Thibault 1845* (RSA), *Hamilton s.n. 17 May 2001* (RSA), *Hirshberg 290* (RSA), *Jones, C. 10* (RSA), *Jones, M.E. 3066* (ARIZ, CAS, DS, POM, UC), *Jones, M.E. s.n. 5 April 1882* (RSA), *Junak, Hoefs, & Crockett SCa-351* (SBBG), *Junak, Hoefs, & Crockett SCa-355* (SBBG), *Junak SCa-361* (SBBG), *Junak, Hoefs, & Crockett SCa-379* (SBBG), *Junak, Hoefs, Takara SCa-399* (SBBG), *Junak, Hoefs, & Stratton SCa-497* (SBBG), *Junak, Hoefs, Takara SCa-514* (SBBG), *Junak & Kirkland SCa-573* (SBBG), *Junak & Kirkland SCa-577* (SBBG), *Junak, Hoefs, Kirkland, & Stratton SCa-631* (SBBG), *Junak, Hoefs, & Kirkland SCa-1439* (SBBG), *Junak SCa-1465* (SBBG), *Junak & Philbrick SCa-1529* (SBBG), *Leatherman 65* (RSA), *Marsh & Marsh s.n. 10 June 1991* (RSA), *Moran & Barber s.n. 8 June 2001* (RSA), Munz & Johnston 5335a* (CAS, POM, UC), ***Palmer 70* (MO; isotype)** *Parikh 156* (SBBG), *Parikh & Gale 1739* (SBBG), *Parish 12060* (CAS), *Parry s.n. 17 March 1882* (DS), *Peirson 3029* (RSA), *Philbrick & Thorne B67-175* (SBBG), *Pringle 269* (CAS), Purer 6927* (UC), Rebman 8031*è (UC), Rebman 8348*è (UC), *Rebman, Gregory, Mulligan, & Ricks 11673* (RSA), Rebman, Gregory, Rich, & Principe 12817* (RSA, UC), *Riefner 20-391* (RSA), *Riefner 20-393* (RSA), *Riefner 95-62* (RSA), *Roberts 3870* (RSA), *Roberts & Bontrager 4565* (RSA), *Roberts, Roberts, & Bontrager 4587* (RSA), *Roberts 4855* (RSA), *Roberts & Bomkamp 4981* (RSA), *Roberts & Bramlet 5563* (RSA), *Roberts & Bramlet 5691* (RSA), Ross 6853* (UC), *Ross 6869* (CAS), *Ross & Takara 6939* (CAS), *Ross, Takara, & Otte 6947* (CAS), *Sanders 26178* (SBBG), *Sanders 32379* (RSA, SBBG), *Sanders, Salvato, Volansky, & Balk 32568* (RSA), *Sanders, Wotipka, Elvin, et al. 26153* (CAS, SBBG), *Thorne 35873* (SBBG), Thorne 35949* (UC), *True 152* (POM), *Vanderwerff 4235* (RSA), *White 8381* (ASU, RSA), *White & Duchardt 8862* (RSA).

## Supplementary Material

XML Treatment for Harpagonella arizonica
